# Engineered disulfide bonds improve thermostability and activity of L‐isoleucine hydroxylase for efficient 4‐HIL production in *Bacillus subtilis* 168

**DOI:** 10.1002/elsc.201900090

**Published:** 2019-10-09

**Authors:** Zhina Qiao, Meijuan Xu, Minglong Shao, Youxi Zhao, Mengfei Long, Taowei Yang, Xian Zhang, Shangtian Yang, Hideki Nakanishi, Zhiming Rao

**Affiliations:** ^1^ The Key Laboratory of Industrial Biotechnology, Ministry of Education, School of Biotechnology Jiangnan University Wuxi Jiangsu Province P. R. China; ^2^ Beijing Key Laboratory of Biomass Waste Resource Utilization, College of Biochemical Engineering Beijing Union University Beijing P. R. China; ^3^ Department of Chemical and Biomolecular Engineering The Ohio State University Columbus OH USA

**Keywords:** 4‐hydroxyisoleucine, *Bacillus subtilis* 168, disulfide bond, l‐isoleucine hydroxylase, molecular dynamics simulation

## Abstract

4‐Hydroxyisoleucine, a promising drug, has mainly been applied in the clinical treatment of type 2 diabetes in the pharmaceutical industry. l‐Isoleucine hydroxylase specifically converts l‐Ile to 4‐hydroxyisoleucine. However, due to its poor thermostability, the industrial production of 4‐hydroxyisoleucine has been largely restricted. In the present study, the disulfide bond in l‐isoleucine hydroxylase protein was rationally designed to improve its thermostability to facilitate industrial application. The half‐life of variant T181C was 4.03 h at 50°C, 10.27‐fold the half‐life of wild type (0.39 h). The specific enzyme activity of mutant T181C was 2.42 ± 0.08 U/mg, which was 3.56‐fold the specific enzyme activity of wild type 0.68 ± 0.06 U/mg. In addition, molecular dynamics simulation was performed to determine the reason for the improvement of thermostability. Based on five repeated batches of whole‐cell biotransformation, *Bacillus subtilis* 168/pMA5‐*ido* ^T181C^ recombinant strain produced a cumulative yield of 856.91 mM (126.11 g/L) 4‐hydroxyisoleucine, which is the highest level of productivity reported based on a microbial process. The results could facilitate industrial scale production of 4‐hydroxyisoleucine. Rational design of disulfide bond improved l‐isoleucine hydroxylase thermostability and may be suitable for protein engineering of other hydroxylases.

**Abbreviations**
4‐HIL: 4‐hydroxyisoleucine
CD: circular dichroism
IDO: l‐isoleucine hydroxylase
K152C: 152 lysine residues of l‐isoleucine hydroxylase protein mutated to cysteine
RMSD: root mean square deviation
RMSF: root mean square fluctuation
*t*_1/2_: half‐life of heat inactivation
T181C: 181 threonine residues of l‐isoleucine hydroxylase protein mutated to cysteine

## 1 INTRODUCTION

4‐Hydroxyisoleucine (4‐HIL) is a natural nonproteinogenic amino acid that was first discovered in fenugreek seeds 1, 2. 4‐HIL is a promising drug with potential applications mainly in the pharmaceutical industry. 4‐HIL has attracted a great deal of attention due to its considerable insulin secretion promotion effects, enhancement of resistance to insulin in peripheral tissues, and dyslipidemia regulation 3. To date, the methods of synthesizing 4‐HIL mainly include plant separation extraction, chemical‐enzyme synthesis, and microbial transformation 4, 5, 6, 7. The plant separation extraction methods have been mainly applied in the extraction of 4‐HIL from fenugreek seeds, but the yields are relatively low (approximately 150 mg of 4‐HIL extracted from 1 kg of fenugreek seeds) 8. Chemical–enzymatic synthesis methods have also been evaluated and are associated with low efficiency, high costs, and severe pollution 5, 6, 7. With the constant increase in environmental awareness and the development of green technologies, nonpolluting and nontoxic biological technologies have inevitably become favored in industrial development 9, 10.

Recently, the use of whole‐cell processing l‐isoleucine hydroxylase (IDO) to specifically catalyze l‐Ile into 4‐HIL has received considerable attention. In addition, *ido* derived from *Bacillus thuringiensis* 2e2, *Bacillus thuringiensis* TCCC 11826, *Bacillus thuringiensis* YBT 1520, and *Bacillus weihenstephanensis* has been expressed in *Escherichia coli* or *Corynebacterium glutamicum*. 4‐HIL can be obtained using biotransformation or direct fermentation 1, 2, 11, 12, 13. However, due to its poor thermostability, IDO enzyme activity decreased considerably when the temperature exceeded the optimal value 1, 2, 14, 15, 16. To date, to the best of our knowledge, no study has explored or reported on the molecular modification of IDO to improve its thermostability.

The methods that have been extensively applied to improve enzyme thermostability include homology alignment, proline design, disulfide bond design, and surface charge optimization 17. The introduction of disulfide bonds into cellobiohydrolase 18, 19, lipase 20, 21, and xylanase 22, 23 considerably improved their thermostability and activity. The introduced disulfide bond stabilizes the protein in a reversibly folded state by decreasing the folding entropy 24 or in an irreversibly folded state by decreasing the folding rate of the protein 25. So, we designed disulfide bonds on the loops to enhance IDO thermostability to facilitate efficient 4‐HIL production.

*Bacillus subtilis*, an industrial workhorse that is widely applied in the large‐scale microbial production of recombinant proteins, amino acids, and fine chemicals 26, 27, is generally recognized as safe by the Food and Drug Administration (FDA) 28, 29, 30. Although *B. subtilis* has been extensively applied in the production of valuable metabolites, bioremediation, and energy generation, there was no study with regard to its application in 4‐HIL production. Therefore, we selected *B. subtilis* 168 as the host that would express IDO for 4‐HIL production.

In the present study, we first expressed *ido* from *Bacillus cereus* 13658 in *B. subtilis* 168. The thermostability and enzyme activity of IDO were increased through the rational design of the disulfide bond. The half‐life of IDO at 50°C increased from 0.39 to 4.03 h, which was a 10.27‐fold increase. In addition, the specific enzyme activity of IDO increased from 0.68 ± 0.06 U/mg to 2.42 ± 0.08 U/mg, which was a 3.56‐fold increase. In addition, we investigated the mechanism of half‐life and specific enzyme activity increase using molecular dynamics simulation.

Finally, the whole cell of *B. subtilis* 168/pMA5‐*ido* ^T181C^ recombinant strain was used for the biotransformation of l‐Ile into 4‐HIL. The five repeated batches gave a cumulative yield of 856.91 mM (126.11 g/L) 4‐HIL, which was 2.19 times higher than the 390.52 mM (57.47 g/L) yield obtained from the wild‐type strain. The results of the present study offer novel insights that could facilitate the enhancement of IDO thermostability and pave the way for the industrial application of 4‐HIL.

**PRACTICAL APPLICATION**

4‐HIL has the effects of promoting insulin secretion, improving the resistance of peripheral tissues to insulin, and regulating dyslipidemia, which is mainly used in the pharmaceutical industry. l‐isoleucine hydroxylase is commonly used to produce 4‐HIL. However, the applicability of l‐isoleucine hydroxylases in industrial applications is restricted by their low thermostability or low activity at high temperature. Therefore, we designed the disulfide bond for l‐isoleucine hydroxylase to improve thermostability. Finally, the obtained optimal mutant strain *Bacillus subtilis* 168/pMA5‐*ido* ^T181C^ rain produced a cumulative yield of 856.91 mM (126.11 g/L) 4‐HIL after five batches, which is the highest level of productivity reported based on a microbial process. This strategy enhanced thermostability and enzyme activity of l‐isoleucine hydroxylase, which potentially be used for large‐scale 4‐HIL industrial production. The approach also may be suitable for protein engineering of other hydroxylases for the efficient production of other valuable hydroxylated amino acids.

## 2 MATERIALS AND METHODS

### 2.1 Strains, plasmids, and materials

*ido* (GenBank: AE016877.1) encoding IDO from *B. cereus* 13658 was used as the template. Plasmid pMA5 was utilized as the expression vector for IDO and its mutants. *E. coli* JM109 was used as the host for *ido* cloning and *B. subtilis* 168 as the expression host for IDO and its variants. PrimeSTAR HS DNA polymerase, restriction enzymes, and T4 DNA ligase were purchased from TaKaRa (Dalian, China).

### 2.2 Construction of wild type strain and its mutants

The *ido* amplified by PCR was ligated to pMA5 plasmid to construct recombinant plasmid pMA5‐*ido*. Site‐directed mutagenesis on the K152 and T181 sites of the *ido* to cysteine was performed by overlap extension PCR 31 using primers listed in Supporting Information Table S1. The 6×His tag was added downstream of the primer to facilitate purification of the recombinant enzymes. The mutated genes (*ido* ^K152C^ and *ido* ^T181C^) were also ligated to pMA5 to generate pMA5‐*ido* ^K152C^ and pMA5‐*ido* ^T181C^. Recombinant plasmids pMA5‐*ido*, pMA5‐*ido* ^K152C^, and pMA5‐*ido* ^T181C^ were transformed into *E. coli* JM109 to obtain recombinant strains *E. coli* JM109/pMA5‐*ido*, *E. coli* JM109/pMA5‐*ido* ^K152C^, and *E. coli* JM109/pMA5‐*ido* ^T181C^, respectively. The recombinant plasmids were also transformed into *B. subtilis* 168 to obtain recombinant strains *B. subtilis* 168/pMA5‐*ido*, *B. subtilis* 168/pMA5‐*ido* ^K152C^, and *B. subtilis* 168/pMA5‐*ido* ^T181C^ (Supporting Information Table S2).

### 2.3 Protein expression and purification

The recombinant strains *B. subtilis* 168/pMA5‐*ido*, *B. subtilis* 168/pMA5‐*ido* ^K152C^, and *B. subtilis* 168/pMA5‐*ido* ^T181C^ were cultured overnight at 37°C in Lysogeny Broth (LB) medium supplemented with 50 µg/mL kanamycin. The cells were then incubated into 50 mL LB medium and cultured at 37°C shaker at 180 rpm for 20–24 h to obtain IDO, K151C, and T181C protein. The cells were harvested by centrifugation at 8000 rpm at 4°C for 10 min (refrigerated centrifuge, Sigma) and then washed twice with phosphate‐buffered saline (PBS) (50 mM, pH 7.0). The harvested cells were added to lysozyme for 1–2 h on ice and sonicated and centrifuged at 10 000 rpm for 30 min at 4°C to remove cell debris. The supernatant was purified and used for the enzyme activity assay.

Wild type and its variants were subjected to metal affinity chromatography on an AKTA purifier system (Amersham Pharmacia Biotech, UK) using a 1‐mL His Trap FF column (GE Life Sciences, USA) with linear gradient elution experiments using 0 to 700 mM imidazole as the elution buffer. The purified wild type and its mutant enzymes were analyzed by SDS‐PAGE (12% acrylamide). Protein concentrations were determined using a Bradford Protein Assay Kit 32.

### 2.4 Enzyme activity assay and 4‐HIL determination

Wild type and its mutants’ enzyme activities were determined using the 2,4‐dinitrofluorobenzene precolumn derivatization method 1. The assay mixture contained 30 mM l‐isoleucine, 30 mM α‐KG, 1 mM FeSO_4_·7 H_2_O, and 5 mM ascorbic acid in PBS buffer (50 mM, pH 7.0) at 30°C for 30 min with 0.1 mg/mL of wild type and its mutants. The reaction solution was boiled in a water bath for 5 min to terminate the enzyme reaction. The reaction solution was subsequently subjected to the derivatization treatment, and the 4‐hydroxyisoleucine concentrations were determined using HPLC. The amount of enzyme that converted l‐Ile into 1 µmol 4‐HIL was defined as 1 unit (U).

### 2.5 Determination of optimum temperatures and pH of the wild type enzyme and its variants

The optimum temperatures for wild type and its mutants were determined by adding 0.1 mg/mL enzymes to a reaction system consisting of 30 mM l‐Ile, 30 mM α‐KG, 1 mM FeSO_4_·7H_2_O, 5 mM ascorbic acid, and PBS buffer (50 mM, pH 7.0). Relative enzyme activity was measured at 20–60°C for 1 h. The optimum pH was determined by adding 30 mM l‐Ile, 30 mM α‐KG, 1 mM FeSO_4_·7H_2_O, 5 mM ascorbic acid, and 0.1 mg/mL of the wild type enzyme and its mutants in buffers with different pH values (3.0–10.0). The reaction was carried out at 30°C for 1 h, and the relative enzyme activity was measured. The different buffers used included the following: 50 mM sodium citrate–citrate buffer (pH 3.0–5.0), PBS buffer (pH 6.0–8.0), Tris‐HCl (pH 9.0), and sodium bicarbonate–sodium carbonate solution (pH 10.0).

The thermostability of wild type and its mutants was determined by incubating them at 10–70°C for 20 min, prior to the addition of 30 mM l‐Ile, 30 mM α‐KG, 1 mM FeSO_4_·7H_2_O, 5 mM ascorbic acid, and PBS buffer (50 mM, pH 7.0). The reaction mixture was incubated at 30°C for 1 h, and the residual enzyme activity was measured. To further examine the thermostability of wild type and its variants, the half‐life (*t*_1/2_) of the wild type and its variants were tested at 50°C. Here, similar concentrations of wild type and its variants were heat treated at 50°C for different time periods, then added to a reaction system consisting of 30 mM l‐Ile, 30 mM α‐KG, 1 mM FeSO_4_·7H_2_O, 5 mM ascorbic acid, and PBS buffer (50 mM, pH 7.0) and reacted at 30°C for 1 h prior to the measurement of the relative enzyme activity. The first‐order rate constant k was obtained from the slope of the incubation time and the semilogarithmic plot of residual activity, and the *t*_1/2_ value at 50°C was calculated using the following equation: *t*_1/2_ = ln2/k. Enzyme incubations were performed in metal bath at a constant temperature to precisely control temperature and incubation time.

The kinetic constants (*K*_m_, *k*_cat_, and *k*_cat_/*K*_m_) were measured under conditions limiting only one substrate (l‐Ile or α‐KG). The *K*_m_ of α‐KG was measured based on a change in the α‐KG concentration from 0.0125 to 40 mM at an l‐Ile concentration of 20 mM, and the l‐Ile concentration was determined based on a change from 0 to 5 mM when the α‐KG concentration was 20 mM. Finally, the corresponding data were substituted in the Michaelis–Menten equation to obtain the kinetic constants *k*_cat_ and *K*_m_ of the two substrates using GraphPad Prism v7.0 (GraphPad Software, Inc., San Diego, CA, USA). All kinetic constants are average values obtained from triplicate measurements.

### 2.6 Verification of disulfide bond formation

To confirm the presence of a disulfide bond in the variant protein, the formation of a disulfide bond was determined by analyzing the concentrations of free cysteine in the protein using dithionitrobenzoic acid as a reagent 33.

### 2.7 Circular dichroism for protein secondary structure analysis

Wild type and its variants were scanned in a quartz cuvette with a diameter of 0.1 cm using a circular dichroism (CD) instrument (MOS‐450/AF‐CD‐STP‐A, Bio‐Logic, Grenoble, France) in the far ultraviolet region in the range of 190–260 nm 34. Ultrapure water was used as the control. The data from the CD scan was processed using the DichroWeb online server (http://dichroweb.cryst.bbk.ac.uk/html/process.shtml) to determine the percentage estimates of the α‐helix, β‐sheet, β‐turn, and random curl contents 35.

### 2.8 Structure and molecular dynamics analysis

The Protein Fold Recognition Server (Phyre2) (http://www.sbg.bio.ic.ac.uk/phyre2/html/page.cgi?id=index) was used to predict the 3D‐structure model 36. The online software SAVES v5.0 (http://servicesn.mbi.ucla.edu/SAVES/) was used to score the predicted 3D‐structure models 37, 38. The Disulfide by Design platform (http://cptweb.cpt.wayne.edu/DbD2/index.php) was used to design disulfide bonds 32. Model observation, image processing, and correlation analyses among amino acid residues were performed using PyMOL (https://pymol.org/2/) and VMD (http://www.ks.uiuc.edu/Research/vmd/) 39, 40, 41. Molecular dynamics simulations were performed using GROMACS (http://www.gromacs.org/) to analyze the thermal fluctuations in IDO and its derivatives at 300 Kz 42. Rosetta software was used to predict protein stability by calculating the relative change in folding free energy ΔΔ*G* 43, 44.

### 2.9 Whole‐cell biotransformation of 4‐HIL

Wild type strain and its mutant *B. subtilis* 168/pMA5‐*ido* ^T181C^ were first streaked on LB agar medium containing 50 µg/mL kanamycin and incubated overnight at 37°C. Single colonies were added to 10 mL LB liquid medium containing 50 µg/mL kanamycin and incubated for 12 h at 37°C. Subsequently, 1% inoculum concentration was transferred into 150 mL LB medium containing 50 µg/mL kanamycin for 12 h at 37°C as the seed solution. Cells were cultured for 20–24 h in a 5‐L fermenter (Bao Xing, China) containing 1.5 L fermentation medium. The fermentation medium (pH 7.0) contained (g/L) glucose 20, tryptone 10, yeast extract 5, KH_2_PO_4_ 0.75, K_2_HPO_4_·3H_2_O 1.25, MgSO_4_·7H_2_O 1. The aeration rate, agitation speed, and temperature were 4.0 vvm, 600 rpm, and 37°C, respectively. pH was maintained at 7.0 using ammonium hydroxide solution (50%, v/v). After fermentation, the cells were centrifuged at 8000 rpm at 4°C for 10 min then washed twice with PBS buffer (50 mM, pH 7.0). The obtained cells were subjected to whole‐cell biotransformation to produce 4‐HIL.

The one‐batch whole‐cell biotransformation approach applied involved dosing of the substrate and its cofactors for 4‐HIL production. Here, 200 mM l‐Ile and α‐KG, 5 mM FeSO_4_·7H_2_O, 10 mM ascorbic acid, and 200 mL PBS buffer (50 mM, pH 7.0) were added into a 500‐mL shake flask, and then 10 g cells and 1 mL of Triton X‐100 were added, successively. The transformation was carried out at 30°C, and samples were obtained every 3 h over a 21‐h period conversion.

Finally, the repeated batches of the recombinant cells were subjected to transformation. Ten grams of recombinant cells were separately added to 200 mL of substrate solution (50 mM pH 7.0 PBS buffer, 200 mM l‐Ile and α‐KG, 5 mM FeSO_4_·7H_2_O, 10 mM ascorbic acid, and 1 mL Triton X‐100) and transformed at 30°C for 9 h. At the time, the l‐Ile in the conversion solution was almost used up, and the recombinant cells were collected by centrifugation to remove the transformation solution. The recombinant cells were placed in a freshly prepared 200 mL substrate solution, and conversion was continued at 30°C for 9 h. The recombinant cells were obtained from five repeated batches.

## 3 RESULTS AND DISCUSSION

### 3.1 Selection of the IDO target residues for mutation

The properties of the pure wild type enzyme were studied. The optimum reaction temperature was 30°C. When the temperature was increased to 40°C, enzyme activity decreased significantly to 52.6% (Supporting Information Figure S1A), which could limit the industrial production of 4‐HIL. Therefore, it is critical to improve the thermostability of IDO using rational modification.

The amino acid sequences of IDO were submitted to Phyre2 to predict the 3D‐structure model. The crystal structure of the protein (PDB: 3pl0B) was used as a template for modeling, and 226 residues (92% of the sequence) were modeled with 100% confidence using the single highest scoring template. The quality of the predicted structure was evaluated using the SAVES v5.0 37, 38. A Ramachandran plot revealed that 97.1% of the residues were in allowed regions and 0.4% in disallowed regions. WHATCHECK, PROVE (55 buried outlier protein atoms, 7.2% error), and ERRAT (Overall Quality Factor A: 87.4) analyses also confirmed that the model was reliable. Therefore, based on all the analytical results, the protein structure of the model was of high quality (Figure S2). The IDO protein has three free cysteines: C61, C112, and C226; therefore, the PDB file of IDO was submitted to Disulfide by Design. The residue K152 was selected to pair with C61 and residue T181 with C226 based on the sizes of dihedral angles (C112 could not be paired). The *B. subtilis* 168/pMA5‐*ido* ^K152C^ and *B. subtilis* 168/pMA5‐*ido* ^T181C^ mutants were obtained.

### 3.2 Properties of wild type and its mutants

**Figure 1:**
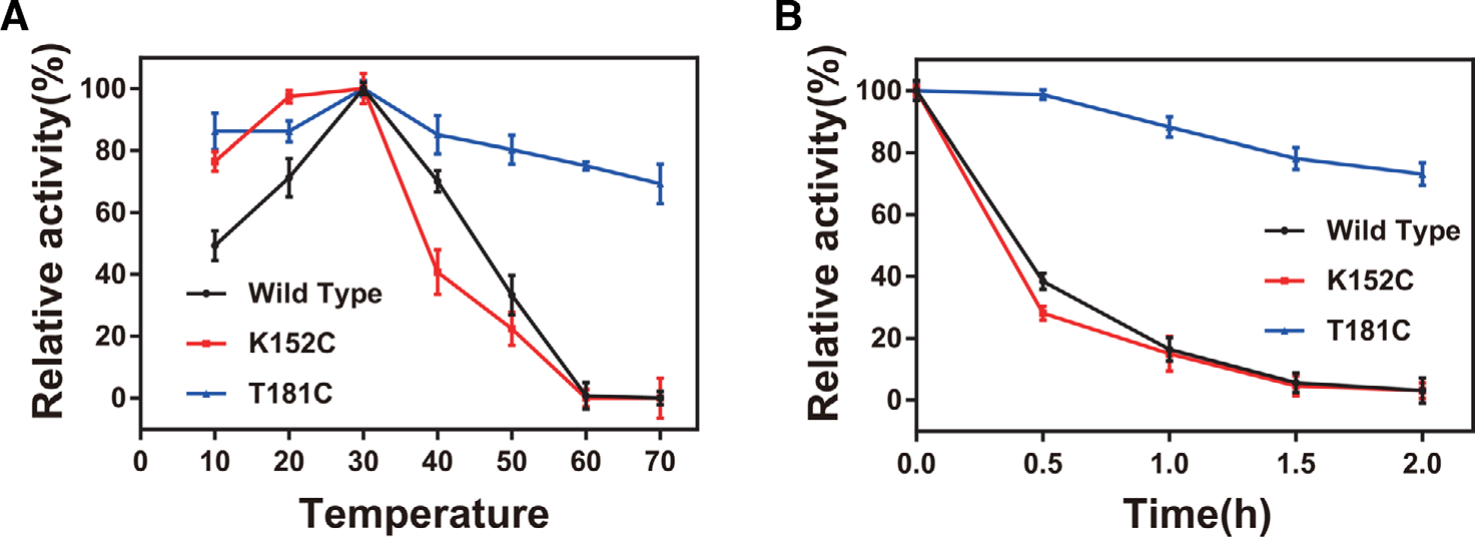
Enzymatic thermostability of wild type and its mutants. (A) Enzyme inactivation assay at 10–70°C for 20 min. (B) Wild type and its mutants heat‐treated at 50°C for different times. All assays were performed in triplicate with three independent measurements. Error bars represent SD of the biological replicates

There were no significant differences in the optimum temperatures (30°C) and pH (7.0) of wild type and its mutants (Supporting Information Figure S1). The relative enzyme activity of T181C (181 threonine residues of l‐isoleucine hydroxylase protein mutated to cysteine) and K152C (152 lysine residues of l‐isoleucine hydroxylase protein mutated to cysteine) remained at 84.3% and 90.3%, respectively, at 40°C, which was an improvement compared with the 52.6% in wild type. To verify their thermostability, wild type and its mutants were incubated at 10–70°C for 20 min to measure residual enzyme activity. T181C retained 69.4% of enzyme activity, whereas wild type and K152C nearly lost enzyme activity (Figure 1A). To further reveal the differences in thermostability, these enzymes were incubated at 50°C for different lengths of time, and the half‐life of heat inactivation (*t*_1/2_) was determined (Figure 1B). According to the results, T181C was considerably more stable compared with both wild type and K152C. In addition, the inactivation process was consistent with the first‐order kinetics (Supporting Information Figure S3), the *t*_1/2_ value of T181C (4.03 h) was 10.27‐fold that of wild type (0.39 h) at 50°C, whereas there was no significant change in the *t*_1/2_ values of K152C (0.40 h). However, the *t*_1/2_ value of the T181C variant was similar to the *t*_1/2_ value of the wild type under high concentrations of dithiothreitol, suggesting that the disulfide bond was destroyed under reducing conditions (data not shown). The results indicated that disulfide bonds play a key role in enhancing IDO thermostability.

The specific activity of T181C was 2.42 ± 0.08 U/mg, which was 3.56‐fold that of wild type, which was 0.68 ± 0.06 U/mg, whereas that of K152C decreased slightly to 0.38 ± 0.05 U/mg. The kinetic properties of wild type and its mutants were determined using l‐Ile and α‐KG as substrates. As shown in Table 1 and Supporting Information Figure S4, in the case of l‐Ile, the *K*_m_ values of K152C and T181C were higher than the *K*_m_ values of wild type, indicating that the affinity for l‐Ile decreased slightly. The (*k*_cat_/*K*_m_)^l‐Ile^ of K152C was only 1.36 s^−1^/mM^−1^, which was considerably lower than that of wild type (3.18 s^−1^/mM^−1^), which could be due to lower catalytic ability of l‐Ile. In addition, the (*k*_cat_/*K*_m_)^l‐Ile^ value of T181C was 1.13‐fold that of wild type, which enhanced the catalytic efficiency of l‐Ile. In the case of α‐KG, the *K*_m_ values of K152C and T181C were lower than that of wild type. Based on the results, the affinity of K152C and T181C for α‐KG increased. The *k*_cat_ of T181C was only 0.14 s^−1^, which was substantially lower than the *k*_cat_ of wild type (0.32 s^−1^), which could have been influenced by the catalytic ability of α‐KG resulting in a further decrease in specific activity. The (*k*_cat_/*K*_m_)^α‐KG^ of T181C was 0.22 s^−1^/mM^−1^, which was 2.44 times higher than that of IDO (0.09 s^−1^/mM^−1^), which enhanced the catalytic efficiency of α‐KG. Based on the typical Fe/αKGD family enzyme structure TauD (PDB: 1GY9) (Figure 3), T220 and R62 are the predicted α‐KG binding sites. The position of the disulfide bond is near the potential α‐KG active site; therefore, it may influence specific activity.

**Table 1:** The kinetic parameters of wild type and its mutants

	l‐ILe			α‐KG		
Enzymes	*K*_m_(mM)	*k*_cat_(s^−1^)	*k*_cat_/*K*_m_(s^−1^/mM^−1^)	*K*_m_(mM)	*k*_cat_(s^−1^)	*k*_cat_/*K*_m_(s^−1^/mM^−1^)
Wild type	0.26	0.84	3.18	3.34	0.32	0.09
K152C	0.90	0.83	1.36	0.90	0.14	0.47
T181C	0.38	0.92	3.59	2.17	0.47	0.22

### 3.3 Detection of disulfide bond number

To verify the introduction of the disulfide bond into K152C and T181C, we used dithionitrobenzoic acid reagent to determine the contents of free cysteine in the protein. Although a disulfide bond was not introduced in the K152C variant, it was introduced into T181C, whereas no disulfide bond was present in wild type (Table 2).

**Table 2:** Results of the free cysteine and deduced disulfide bond number of the mutants

Enzymes	Free cysteine concentration （µmol/mg protein）	Number of free cysteine (1 molecule protein)	Number of cysteine in protein sequence (1 molecule protein)	Number of disulfide bonds (1 molecule protein)
Wild type	0.115 ± 0.003	3	3	0
K152C	0.126 ± 0.005	4	4	0
T181C	0.068 ± 0.002	2	4	1

### 3.4 Structure and molecular dynamics analysis

CD spectroscopic analysis was used to evaluate the effect of the disulfide bond on the IDO secondary structure. The far‐UV CD (190 to 260 nm) of the wild type and its mutants revealed a series of similar curves (data not shown) 34, 45. Analysis of the protein CD spectra on the DichroWeb server revealed no significant difference between the secondary structure of wild type and the K152C variant (helix, strand, turns, and unordered) 35. Rosetta software analysis showed that the Δ*G*2 for T181C was 71.51 kJ/mol, and the Δ*G*1 for the wild type IDO was 76.16 kJ/mol. The mutant T181C has a negative ΔΔ*G* (ΔΔ*G* = Δ*G*2–Δ*G*1 = −4.65 kJ/mol). The results showed that the introduction of disulfide bond resulted in decreasing the overall folding free energy, thereby improving the thermostability of IDO.

**Figure 2:**
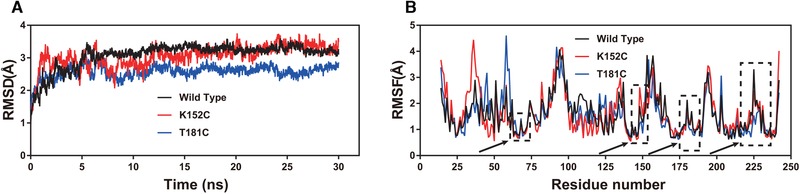
Molecular dynamics analysis of wild type and its variants. (A) RMSD value analysis. (B) RMSF value analysis

**Figure 3:**
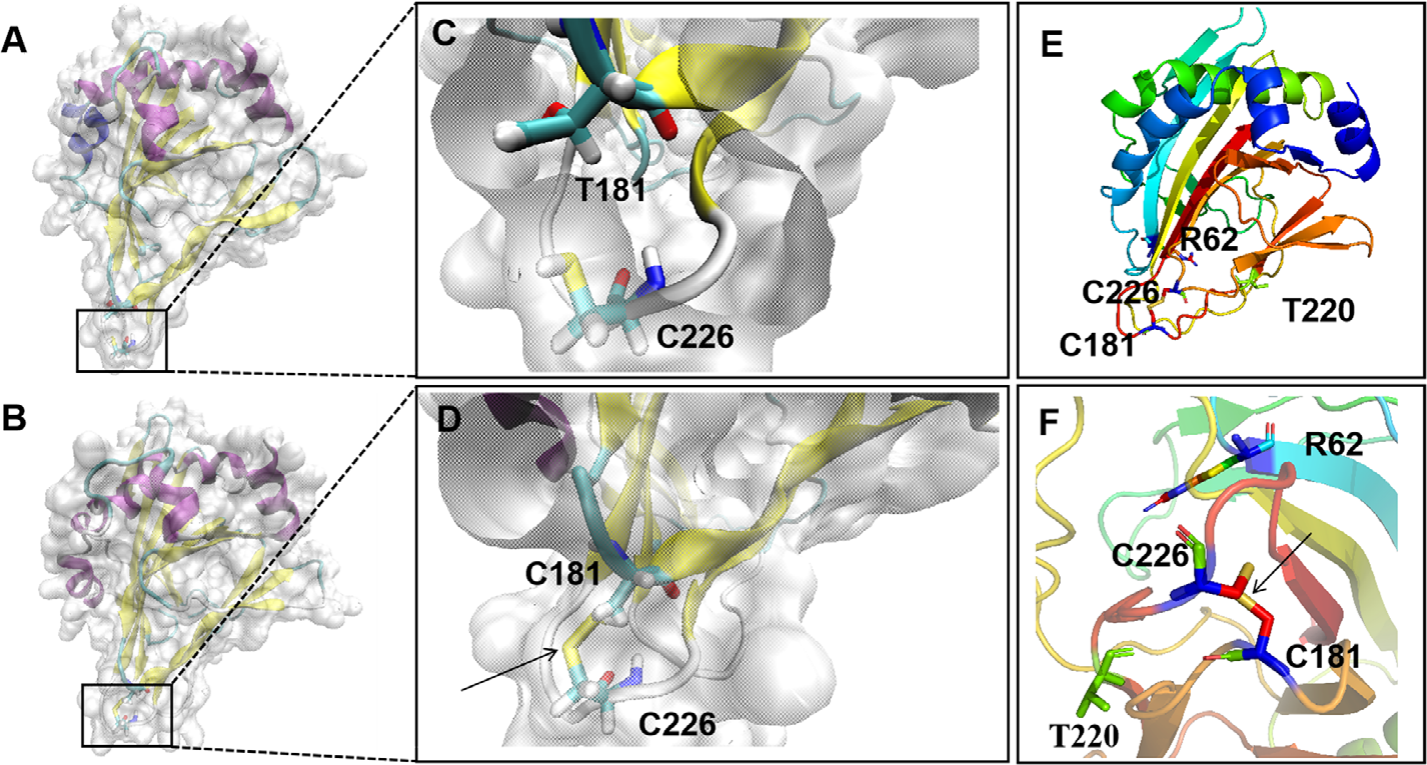
Structural analysis of wild type and its mutant T181C. (A) Structure of wild type. (B) Structure of mutant T181C. (C) Local region of T181 site and C226 site in the tertiary structure of wild type. (D) Local region of C181 site and C226 site in the tertiary structure of T181C. (E,F) Protein structure of T181C containing α‐KG potential binding sites. Purple indicates the α‐helix, yellow indicates the β‐fold, and light blue indicates the loop. The T181, C226, and C181 residues are displayed in the form of sticks, and the disulfide bonds are displayed as yellow sticks and marked with arrows

**Figure 4:**
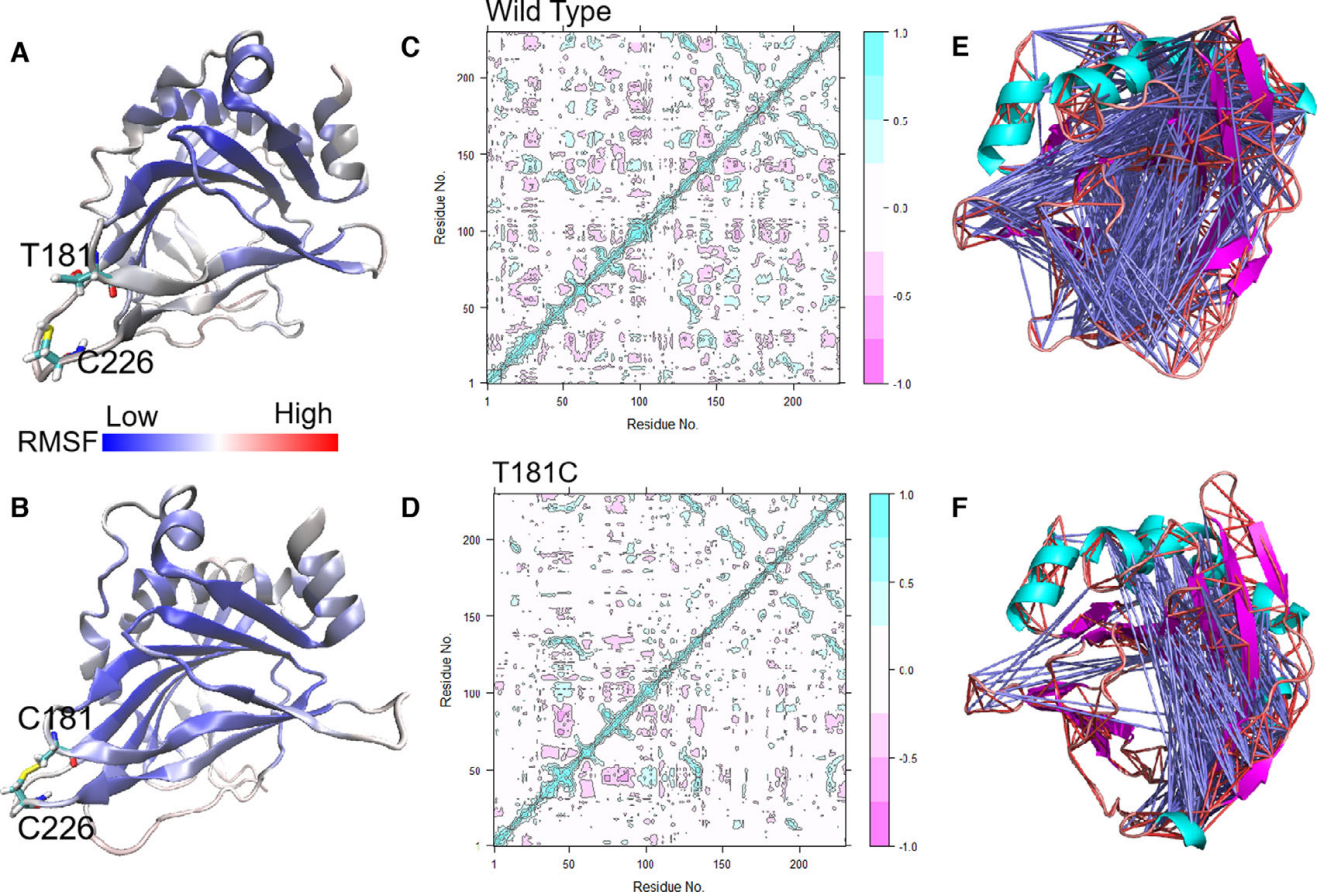
Correlation analysis among residues of all amino acids. (A,B) The RMSF visual analysis of all atoms in the structure of wild type and T181C. Blue and red represent low to high RMSF values, respectively. (C,D) Dynamics cross‐correlation map for the C^α^ atom pairs in IDO and T181C monomers. Correlation coefficients (C_ij_) are presented by different colors. C_ij_ with values from 0 to 1 are positive correlations, whereas C_ij_ with values from −1 to 0 are negative correlations. (E,F) Motion correlation analysis between residues. The blue line indicates the irrelevance between amino acid residues. (E) Motion correlation analysis between residues of wild type. (F) Motion correlation analysis between residues of T181C

Afterward, we simulated the molecular dynamics of wild type and its mutants at 300 K for 30 ns. As shown by the root mean square deviation (RMSD) calculated using the C atom (Figure 2A), all the enzymes exhibited an equilibrium state in the last approximately 13 ns. Generally, an overall decrease in RMSD reflects increased rigidity and in turn increased stability of the enzyme structure 32, 43. The T181C variant had a lower RMSD value than wild type, indicating a more stable structure. With the root mean square fluctuation (RMSF) as an indicator (Figure 2B), variant T181C had reduced RMSF values at regions 178–187 and 223–231, which increased the stability of loops at C181 and C226, which in turn increased the overall stability of wild type. The RMSF values of wild type in the C61 and K152 region were generally low, indicating the relative stability of the regions. Therefore, mutation did not substantially influence the thermostability at position 152 of IDO. Finally, an RMSF visual analysis of all the atoms in the structure wild type and T181C was performed using VMD (Figure 3). For T181C, VMD results showed that loops T181 and C226 were bound tightly, which decreased the flexibility of the loop; The RMSF value decreased near regions T181 and C226, which made the overall enzyme structure more compact and increased the rigidity and stability of the structure. Additional evidence of increased structural rigidity was an improvement in the correlation movement 40, 41 (Figure 4). Pairwise cross‐correlation coefficients (C_ij_) indicate the extent to which the fluctuation of an atom is correlated (or anticorrelated) with another atom, and dynamics cross‐correlation maps show the correlation coefficients (C_ij_) between all C^α^ atom pairs. T181C has less noncorrelation between the amino acid residues compared to wild type, and the correlation between the residues at the 181 and 226 specific sites was increased. The results indicated that the structural stability of the loop region plays a key role in the thermostability of IDO. Therefore, the key role of the C181–C226 disulfide bond in IDO protein thermostability was also supported by the results of molecular dynamics simulation.

### 3.5 4‐HIL synthesis by whole cells expressing IDO and T181C

**Figure 5:**
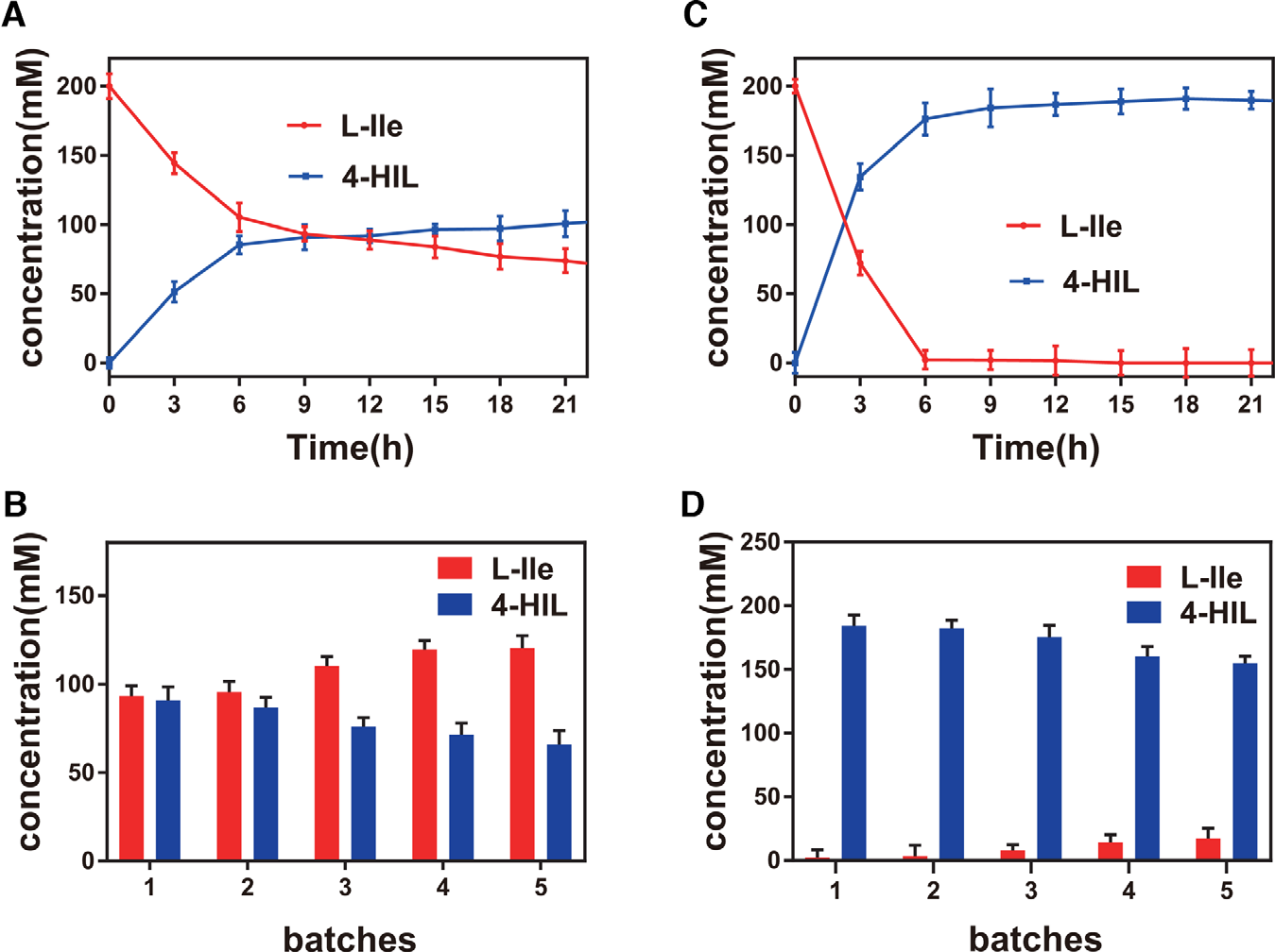
Whole‐cell biotransformation of wild type strain and the *B. subtilis* 168/pMA5‐*ido* ^T181C^ to produce 4‐HIL. (A) One‐batch biotransformation of wild type strain to produce 4‐HIL. (B) One‐batch biotransformation of mutant strain *B. subtilis* 168/pMA5‐*ido* ^T181C^ to produce 4‐HIL. (C) Five repeated batches of wild type strain to produce 4‐HIL. (D) Five repeated batches of mutant strain *B. subtilis* 168/pMA5‐*ido* ^T181C^ to produce 4‐HIL

In this study, one‐batch whole‐cell biotransformation process was applied to *B. subtilis* 168/pMA5‐*ido* ^T181C^ recombinant cells to transform l‐Ile to 4‐HIL at 30°C and pH 7.0. The 4‐HIL yield increased rapidly within the first 3 h, reaching 134.34 ± 9.68 mM, and the productivity was 6.59 g/(L·h). After 9 h, the 4‐HIL yield increased gradually. After 21 h, the final yield reached 190.88 ± 5.57 mM, which was a 1.84‐fold increase compared with the yield from wild type strain (103.84 ± 5.77 mM) (Figure 5).

Based on the above results, the optimal conversion time was determined to be 9 h for the following repeated batch biotransformation. The *B. subtilis* 168/pMA5‐*ido* ^T181C^ cells were used for five batches. The 4‐HIL yield in the fifth batch was still 84.1% that of the first batch (Figure 5D). After the fifth batch, the final cumulative 4‐HIL yield reached 856.91 mM (126.11 g/L), which was a 2.19‐fold increase compared with the yield in wild type strain (390.52 mM, 57.47 g/L) (Figure 5C). The results indicated that the introduction of disulfide bonds enhanced the thermostability and enzyme activity of wild type, hence improved the conversion rate of l‐Ile to 4‐HIL.

Previously, *E. coli* 2Δ (Δ*sucAB*, Δ*aceAK*, P _L_‐*brnQ*) was engineered for synthesis of 4‐HIL by deleting *zwf*, *edd*, and *eda* genes and expressing *B. thuringiensis* 2e2 *ido* gene resulting in approximately 156 mM 4‐HIL (22.9 g/L) from 200 mM supplemented l‐Ile and self‐produced α‐KG 48. The *ido* gene was introduced into *C. glutamicum* strain SN01 producing l‐Ile to synthesize 4‐HIL from its own produced l‐Ile and α‐KG 46. Then, *ppc* was overexpressed to enhance OAA supply, resulting in production 95.72 mM of 4‐HIL after 144 h 15. Quite recently, dynamically modulated the activity of ODHC by l‐Ile‐responsive transcription or attenuation strategies, which produced up to 34.21 g/L 4‐HIL after 64 h of fermentation 13. By synergistically promoting the supplies of three substrates (l‐Ile, α‐KG, and O_2_) and IDO activity, 112–117 mM 4‐HIL was obtained after 72 h fermentation in the optimized medium 47. The main disadvantage of producing 4‐HIL by fermentation is that the cycle is long yet the yield is not very high.

Therefore, the production of 4‐HIL by biocatalytic has become a trend. By deleting the *sucA* and *aceA* genes and expressing a mutant *ido* gene in *E. coli*, 151.9 mM 4‐HIL (22.4 g/L) was synthesized in 12 h by frozen resting cells when 160 mM l‐Ile and 160 mM α‐KG were added 1. Expression of a mutant *ido* gene of *Bacillus weihenstephanensis* in *E. coli*, yielded 66.5 mM 4‐HIL in 24 h by resting cells when 100 mM l‐Ile and 100 mM α‐KG were added 2.Comparing these previous reports with the final cumulative 4‐HIL yield obtained 856.91 mM (126.11 g/L) in this study; we concluded that, it is the highest level of productivity reported based on microbial process. Cell immobilization strategy could be explored at a later stage for industrial production of 4‐HIL.

## 4 CONCLUDING REMARKS

In the present study, we improved the thermostability and enzyme activity of IDO by the rational design of the disulfide bond. The maximum productivity of whole‐cell transformation was increased from 2.53 to 6.59 g/(L·h) after mutation, and the final yield increased from 103.84 ± 5.77 to 190.88 ± 5.57 mM, which is the highest productivity ever reported. The whole cell was continuously transformed into five batches of biocatalyst. The cumulative yield of 4‐HIL from *B. subtilis* 168/pMA5‐*ido* ^T181C^ reached 856.91 mM (126.11 g/L) at 45 h, which was a 2.19‐fold increase compared with the yield of *B. subtilis* 168/pMA5‐*ido* (390.52 mM, 57.47 g/L). Therefore, the results of the present study could facilitate the industrial production of 4‐HIL. This work provides novel insights into the redesigned microreactor, which efficiently produces 4‐HIL based on disulfide bond engineering. The approach also paves the way for the potential enhancement of thermostability and enzyme activity of other hydroxylases for the efficient production of other valuable hydroxylated amino acids.

## CONFLICT OF INTEREST

The authors have declared no conflict of interest.

## Supporting information

FigureS1

FigureS2

FigureS3

FigureS4

Supporting Material
