# Supplementary figures and images for: Engineered disulfide bonds improve thermostability and activity of L‐isoleucine hydroxylase for efficient 4‐HIL production in Bacillus subtilis 168
Source: Eng Life Sci. 2019 Oct 9;20(1-2):7–16. doi: 10.1002/elsc.201900090 (PMC6999076; doi:10.1002/elsc.201900090)

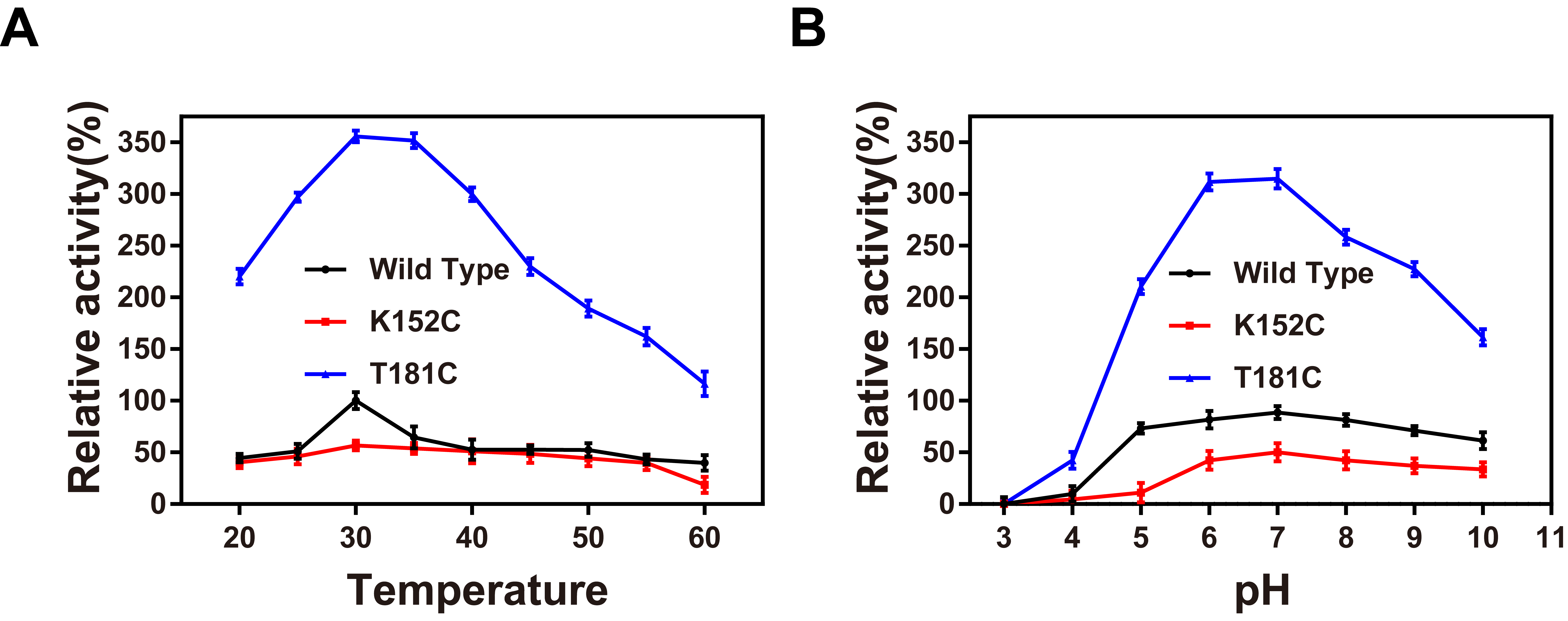

Supplement: Supplementary file 1 — FigureS1 [file ELSC-20-7-s005.tif]

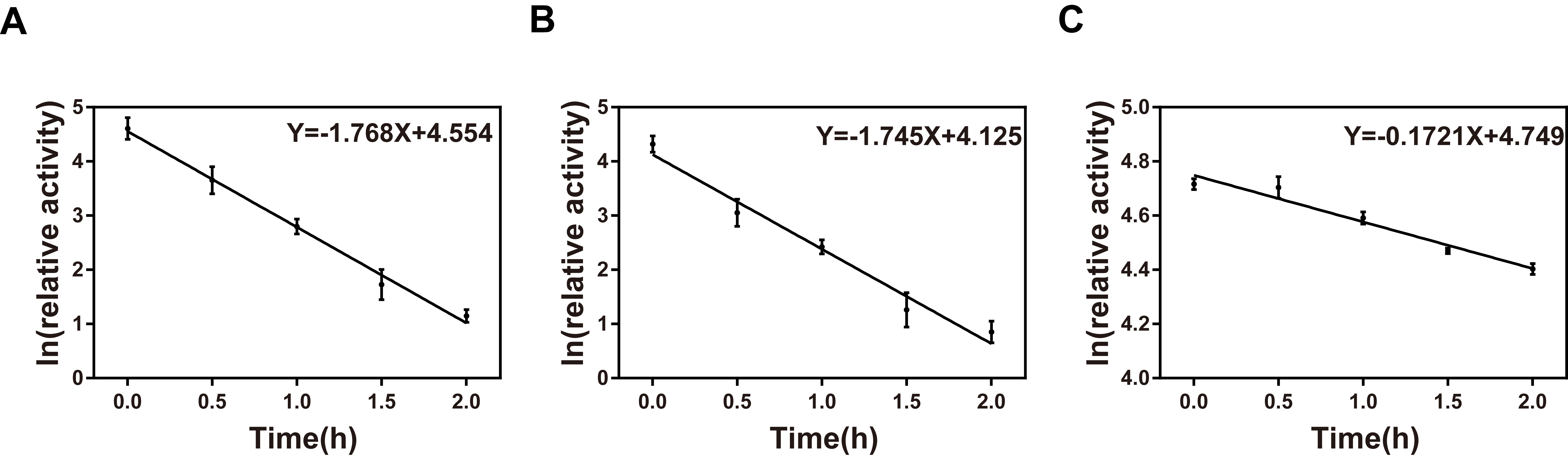

Supplement: Supplementary file 3 — FigureS3 [file ELSC-20-7-s002.tif]

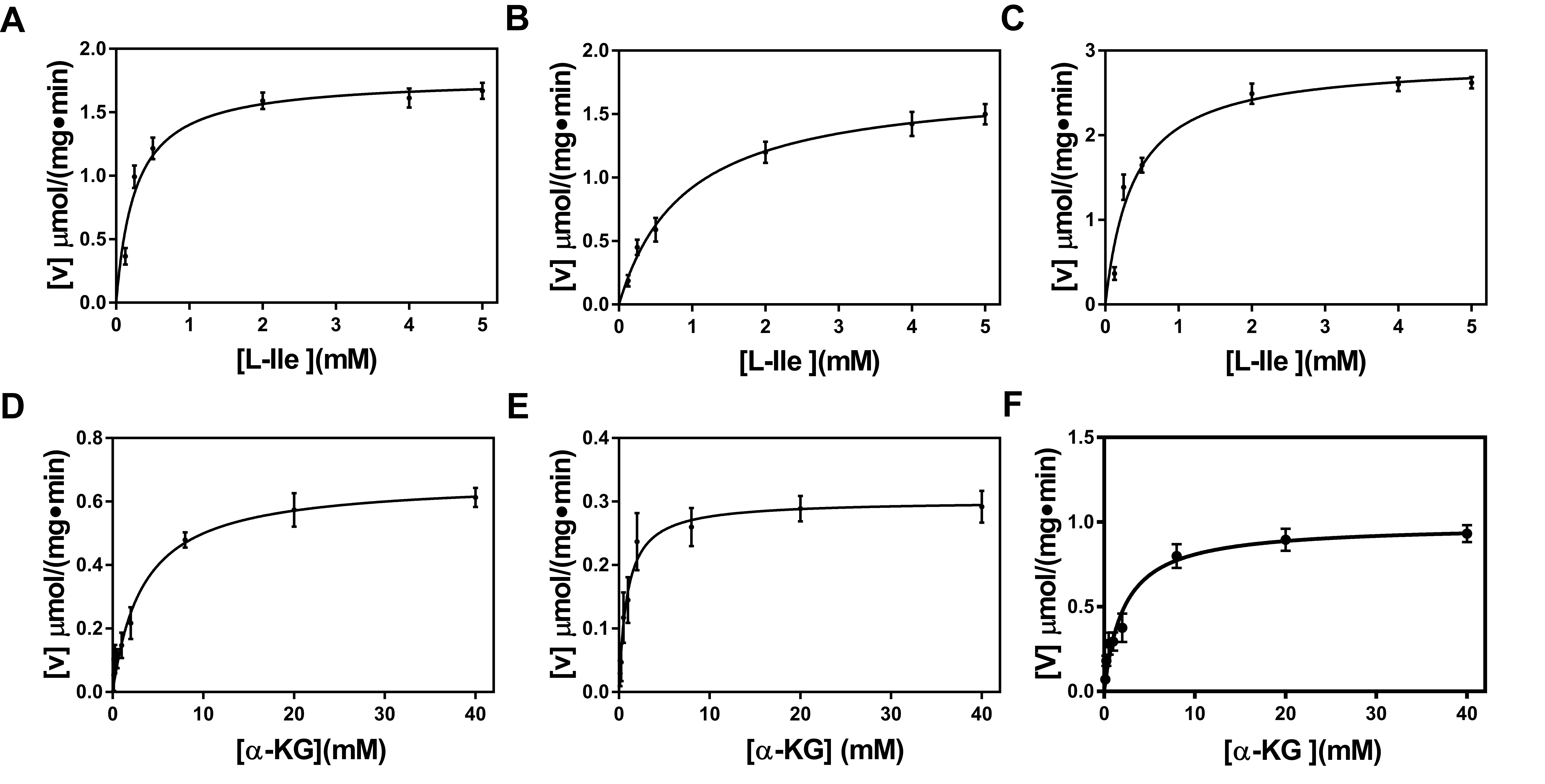

Supplement: Supplementary file 4 — FigureS4 [file ELSC-20-7-s003.tif]
