# Supplementary material for: Engineered disulfide bonds improve thermostability and activity of L‐isoleucine hydroxylase for efficient 4‐HIL production in Bacillus subtilis 168
Source: Eng Life Sci. 2019 Oct 9;20(1-2):7–16. doi: 10.1002/elsc.201900090 (PMC6999076; doi:10.1002/elsc.201900090)
Supplement: Supplementary file 5 — Supporting Material [file ELSC-20-7-s004.docx]

**Supplementary materials**

**Engineered disulfide bonds improve thermostability and activity of L-isoleucine hydroxylase for efficient 4-HIL production** **in *Bacillus subtilis* 168**

Zhina Qiao^a§^, Meijuan Xu^a§^, Minglong Shao^a^, Youxi Zhao^b^, Mengfei Long^a^, Taowei Yang^a^, Xian Zhang^a^, Shangtian Yang^c^, Nakanishi H^a^*, Zhiming Rao^a^*

^a^ The Key Laboratory of Industrial Biotechnology, Ministry of Education, School of Biotechnology, Jiangnan University, Wuxi, Jiangsu Province 214122, China

^b^ Beijing Key Laboratory of Biomass Waste Resource Utilization, College of Biochemical Engineering, Beijing Union University, Beijing, 10023，China

^c^ Department of Chemical and Biomolecular Engineering, The Ohio State University, Columbus, OH 43210, USA

^§^These authors contributed equally to this work.

*Corresponding author at: Jiangnan University, School of Biotechnology, 1800 Lihu Road, Wuxi, Jiangsu 214122, China.

Email address: raozhm@jiangnan.edu.cn (Zhiming Rao).

[hidekinakanishi@hotmail.com](mailto:hidekinakanishi@hotmail.com) (Nakanishi H).

**Table S1** Primers used for gene cloning and site-directed mutagenesis

| Primers | Primer sequences 5’-3’ |
| --- | --- |
| PF: *ido* | AAAAGGAGCGATTTACATATGATGACCTTTGTGCTGAGCAAAA (*Nde* I) |
| PR:ido | ATTTCGACCTCTAGAACGCGTTTAGTGGTGGTGGTGGTGGTGTTTGGTTTCTTTGTAGCTGAAGGTCACCAGTAAAATG (*Mlu* I) |
| PF_m_: K152C | CGCTATAAAGCCACC***TGT***GAACGCCCGAGCTTC |
| PR_m_: K152C | GAAGCTCGGGCGTTC***ACA***GGTGGCTTTATAGCG |
| PF_m_: T181C | ATGAATTTAAGCAAC***TGT***GCCATTGGCGGCGAT |
| PF_m_: T181C | ATCGCCGCCAATGGC***ACA***GTTGCTTAAATTCAT |

Notes: Restriction sites are underlined. Mutation sites are shown in bold and italic. PF_m_ is the upstream of the mutant primer; PR_m_ is the downstream of the mutant primer

**Table S2** Strains and plasmids

| Strains and plasmids | Relevant characteristics | source |
| --- | --- | --- |
| Strains |  |  |
| *Bacillus cereus* 13658 | strain containing *ido* gene | Laboratory store |
| *E. coli* JM109 | gene cloning host | Laboratory store |
| *Bacillus subtilis* 168 | gene expressing host | Laboratory store |
| *E. coli* JM109/pMA5-*ido* | *E. coli* JM109 harboring pMA5-*ido* | This study |
| *E. coli* JM109/pMA5-*ido*^K152C^ | *E. coli* JM109 harboring pMA5-*ido*^K152C^ | This study |
| *E. coli* JM109/pMA5-*ido*^T181C^ | *E. coli* JM109 harboring pMA5- *ido*^T181C^ | This study |
| *B. subtilis* 168/pMA5-*ido* | *B. subtilis* 168 harboring pMA5- *ido* | This study |
| *B. subtilis* 168/pMA5-*ido*^K152C^ | *B. subtilis* 168 harboring pMA5- *ido*^K152C^ | This study |
| *B. subtilis* 168/pMA5-*ido*^T181C^ | *B. subtilis* 168 harboring pMA5- *ido*^T181C^ | This study |
| Plasmids |  |  |
| pMA5 | *E. coli*-*B. subtilis* shuttle plasmid | Laboratory store |
| pMA5-*ido* | pMA5 containing *ido* | This study |
| pMA5-*ido*^K152C^ | pMA5 containing *ido*^K152C^ | This study |
| pMA5-*ido*^T181C^ | pMA5 containing *ido*^T181C^ | This study |


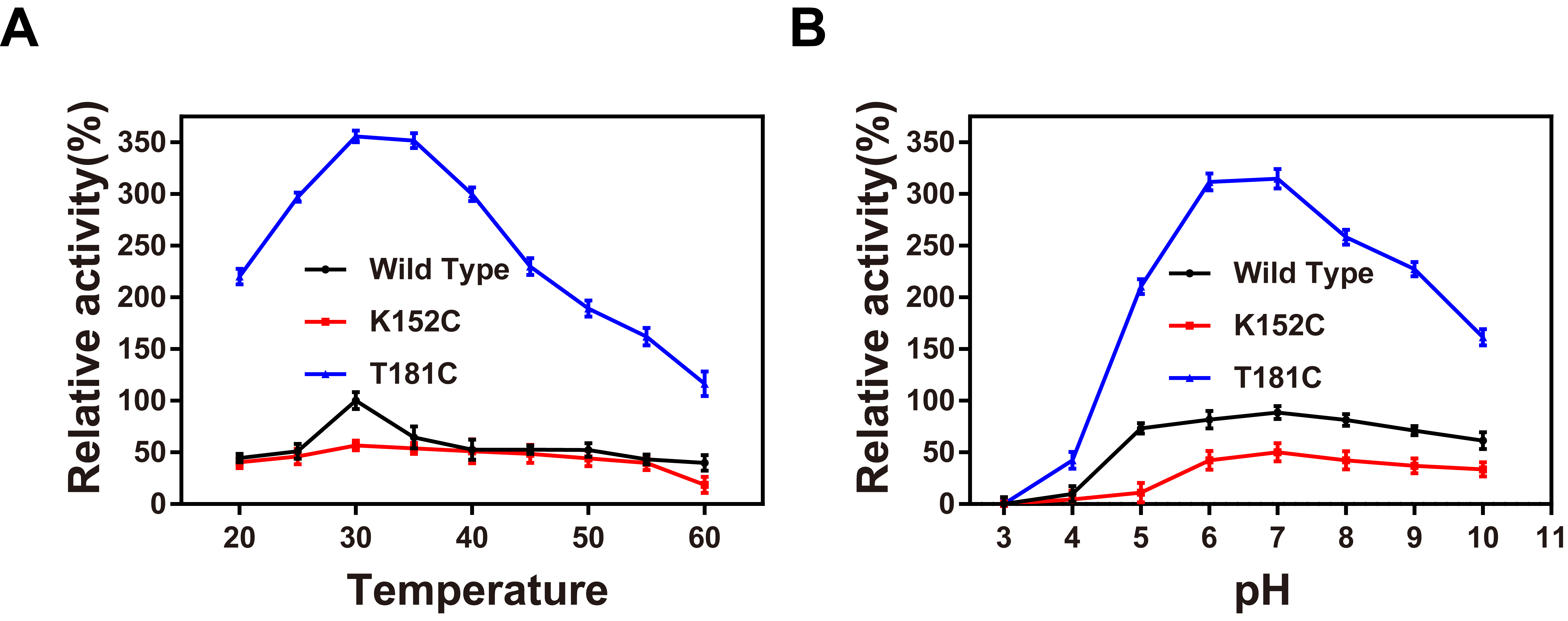


**Figure S1**. **A.** The optimum temperature of wild type and its mutants. **B.** The optimum pH of wild type and the mutant enzymes. All assays were performed in triplicate with three independent measurements. Standard deviations of the biological replicates are represented by error bars.


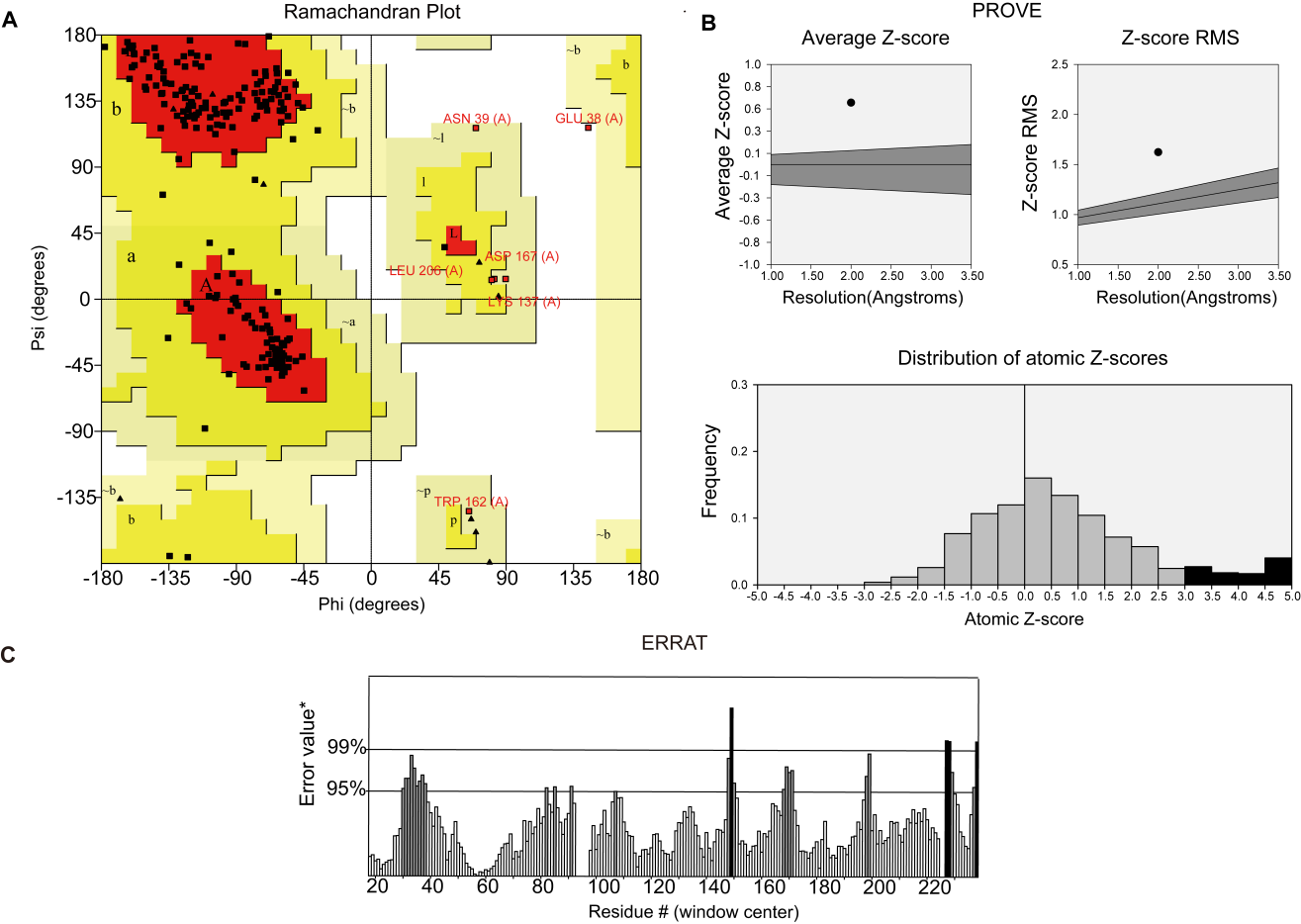


**Figure S2** Results of protein model evaluation. **A**. Ramachandran plot analysis of IDO. The 97.1% of residues were in favored and additional allowed regions. **B.** PROVE analysis of IDO. Its Z-score mean is 0.169, Z-score standard deviation is 1.502, and Z-score of RMS is 1.624. Among the 768 scored atoms, 55 buried outlier protein atoms accounted for 7.2%. **C.** ERRAT analysis of IDO. Overall Quality Factor A is 87.4.


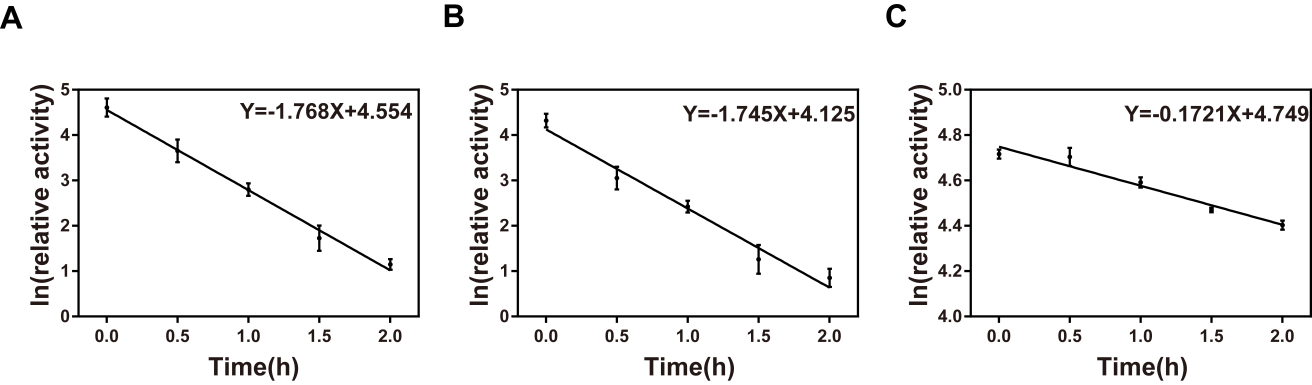


Figure S3 Relationship between time and ln value of residual enzyme activity of wild type and its mutants treated at 50°C for different periods. A. Relationship between time and ln value of residual enzyme activity of wild type. B. Relationship between time and ln value of residual enzyme activity of K152C. C. Relationship between time and ln value of residual enzyme activity of T181C. All assays were performed in triplicate with three independent measurements. Standard deviations of the biological replicates are represented by error bars.


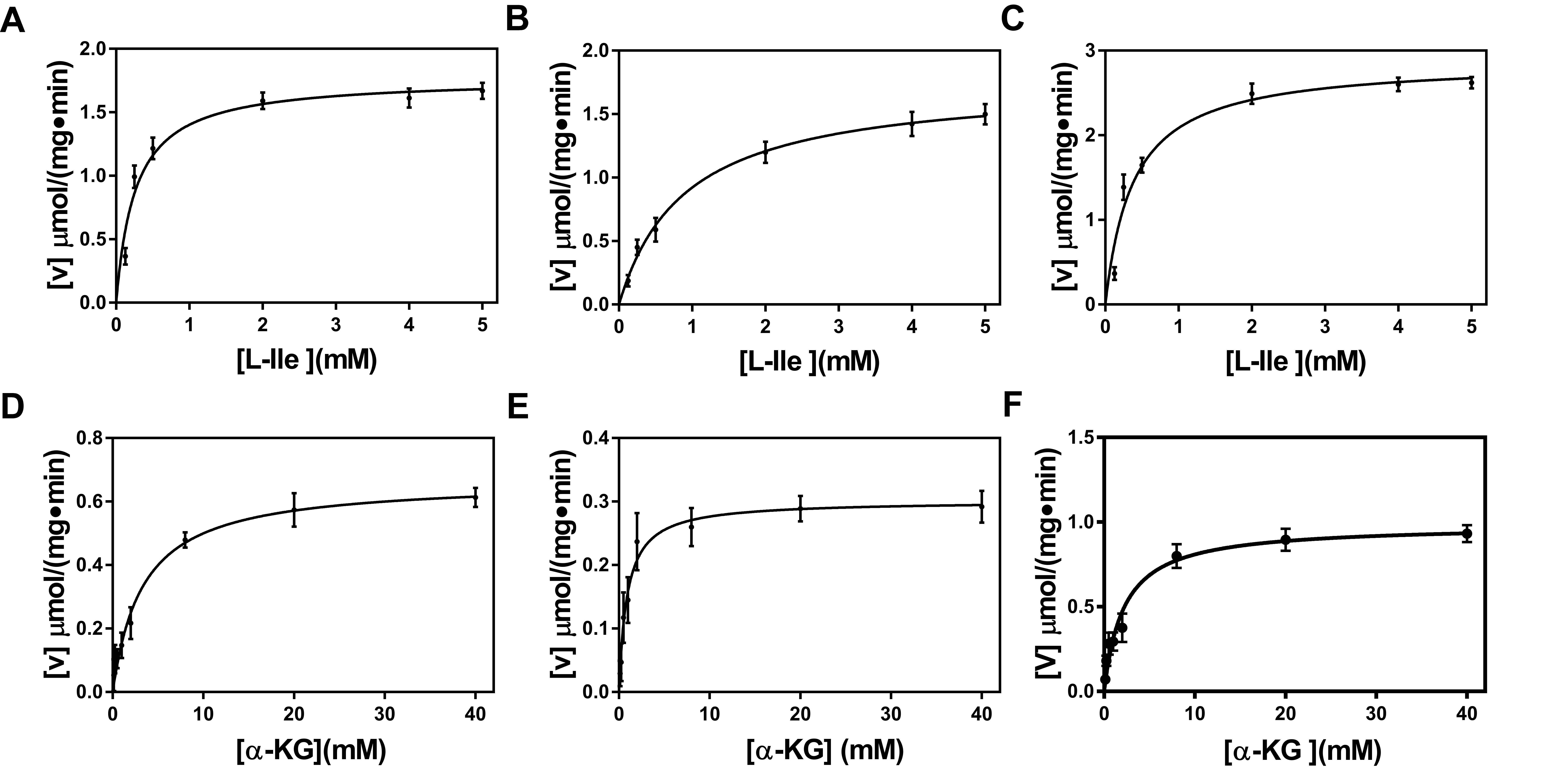


Figure S4 Determination of Michaelis–Menten curve of wild type and its mutant enzymes on substrates L-Ile and α-KG A. Wild type Michaelis–Menten curve of L-Ile B. K152C Michaelis–Menten curve of L-Ile C. T181C Michaelis–Menten curve of L-Ile D. Wild type Michaelis–Menten curve of α-KG E. K152C Michaelis–Menten curve of α-KG F. T181C Michaelis–Menten curve of α-KG.All assays were performed in triplicate with three independent measurements. Standard deviations of the biological replicates are represented by error bars.
